# Care delivery pathways for Chronic Obstructive Pulmonary Disease in England and the Netherlands: a comparative study

**DOI:** 10.5334/ijic.811

**Published:** 2012-05-18

**Authors:** Cecile M.A Utens, J.A.M Maarse, Onno C.P van Schayck, Boudewijn L.P Maesen, Maureen P.M.H Rutten, Frank W.J.M Smeenk

**Affiliations:** Department of Respiratory Medicine, Catharina-hospital Eindhoven, P.O. Box 1350 5602 ZA Eindhoven, The Netherlands; Department of Health, Organization Policy and Economics, School for Public Health and Primary Care (CAPHRI), Maastricht University, P.O. Box 666 6200 MD Maastricht, The Netherlands; Department of General Practice, School for Public Health and Primary Care (CAPHRI), Maastricht University, P.O. Box 666 6200 MD Maastricht, The Netherlands; Department Respiratory Medicine, Orbis Medical Centre, P.O. Box 5500 3130 MB Sittard, The Netherlands; – van Mölken PhD, Institute for Medical Health Technology Assessment, Erasmus University, P.O. Box 1738 3000 DR Rotterdam, The Netherlands; Department of Respiratory Medicine, Catharina-hospital Eindhoven, P.O. Box 1350 5602 ZA Eindhoven, The Netherlands

**Keywords:** comparative analysis, COPD, exacerbations, care delivery pathways, hospital-at-home, integrated care

## Abstract

**Introduction:**

A remarkable difference in care delivery pathways for Chronic Obstructive Pulmonary Disease (COPD) is the presence of hospital-at-home for COPD exacerbations in England and its absence in the Netherlands. The objective of this paper is to explain this difference.

**Methods:**

Descriptive COPD statistics and care delivery pathways on all care levels within the institutional context, followed by a comparison of care delivery pathways and an explanation of the difference with regard to hospital-at-home.

**Results:**

The Netherlands and England show broad similarities in their care delivery pathways for COPD patients. A major difference is the presence of hospital-at-home for COPD exacerbations in England and its absence in the Netherlands. Three possible explanations for this difference are presented: differences in the urgency for alternatives (higher urgency for alternative treatment models in England), the differences in funding (funding in England facilitated the development of hospital-at-home) and the differences in the substitution of tasks to nurses (substitution to nurses has taken place to a larger extent in England).

**Discussion and Conclusion:**

The difference between the Netherlands and England regarding hospital-at-home for COPD exacerbations can be explained in three ways. Hospital-at-home has proved to be a safe alternative for hospital care for selected patients, and should be considered as a treatment option for COPD exacerbations in the Netherlands.

## Introduction

Chronic Obstructive Pulmonary Disease (COPD) is a chronic illness with pulmonary symptoms of dyspnoea, cough and sputum production [1]. The disease is progressive over time and, as yet, not curable [1]. In Western countries the main risk factor for COPD is smoking [1]. The prevalence of COPD is increasing, mainly because of aging and the late effects of smoking [2]. Although high prevalence is a global phenomenon, national and even regional differences can be observed [3, 4]. The burden of the disease is high for patients and society. Health care costs for COPD are rising while available resources are under increasing pressure. Hospital treatment of acute exacerbations of COPD is the main contributor to the high health care costs of COPD [5]. These developments have focused global attention on the disease and have led to new organisational interventions to manage the impact of the disease on patients and society.

Countries often respond in different ways to similar problems. International comparative research can yield insight into these similarities and differences, which in turn can serve to improve health and the functioning of health services [6]. Both the Netherlands and England are facing an increasing prevalence of COPD. Reported prevalence in England varies from 2.5% to 13.3%, depending on the study population and the definitions used [4, 7]. Prevalence is higher in socially deprived areas like the north of England [4]. It is estimated that 31% of patients are undiagnosed [8]. COPD prevalence in the Netherlands varies from 1.9% to 11.6% [3, 7]. The percentage undiagnosed patients is estimated at 19% [9].

A remarkable difference between England and the Netherlands in the organisation of care delivery to COPD patients concerns the development of a new form of care for COPD exacerbations in England—termed ‘hospital-at-home’. Hospital-at-home refers to treatment schemes that substitute (parts of) hospital admission with treatment at home. This arrangement is only available for patients for whom admission is considered necessary. Treatment at home is always for a limited time. In the Netherlands a similar development for patients who need to be admitted has not occurred.

The purpose of this article is to explain why hospital-at-home is present in England and absent in the Netherlands. To do so we will first briefly compare COPD care in England and the Netherlands. Next, we present three complementary explanations for the presence of hospital-at-home in England and its absence (so far) in the Netherlands. Lastly, we address the question of what the Netherlands can learn from England with regard to COPD care.

## Methods

Our study is a descriptive comparison between COPD care in England and the Netherlands based on previous findings published in literature and databases. Statistics on COPD, health care use (i.e. number of hospitalisations and length of stay) and health care resources (i.e. number of respiratory physicians, number of general practitioners, number of available hospital beds and financial resources) were obtained from both national and international databases to guarantee comparability. Furthermore, publications in national and international journals, communications from national professional associations and the results of the Confronting COPD surveys in the Netherlands and the UK performed in 2003 [8, 9] were used. Hospital admissions include admissions with principal International Classifications of Disease codes (ICD-10) J40-J44 [(chronic) bronchitis, emphysema, chronic obstructive pulmonary diseases] and J47 (bronchiectasis) [10]. These generally pertain to admissions for exacerbations. Statistics are presented as figure per 1000, 10,000 or 100,000 population. For the description and comparison of care delivery pathways for COPD patients we studied national and international guidelines on COPD care as well as policy reports and documents from professional associations and organisations involved in COPD care in both countries.

In the UK the responsibility for health (care) is devolved to England, Scotland, Wales and Northern Ireland. This article focuses on England. However, if no other data are available, we use statistics representing the entire UK.

## Results

### Statistical overview of COPD in England

In 1998, the number of annual hospital episodes for COPD was 22 per 10,000 population, which increased to 25 per 10,000 population in 2008 [11]. The number of hospital admissions, standardised for the age distribution of the population, was 360 per 10,000 population in 2007 [11]. In 2003, approximately 14% of COPD patients was hospitalised [8]. The average length of hospital stay for COPD decreased between 1998 and 2008 from 10.2 days to 8.0 days [11]. The Confronting COPD survey showed that the average annual direct health care costs of COPD in 2003 were £819 (€1270) per patient, half of which was spent on hospital admissions [8]. About 53% of COPD patients have at least one comorbidity besides their COPD [8] and 55% of patients use some (inhaled) medication for their COPD [8].

### Statistical overview of COPD in the Netherlands

Between 1998 and 2002, the number of annual hospital episodes for COPD dropped from 14.9 to 10.9 admissions per 10,000 populations. Since then the number has remained stable [12]. In 2007, the number of hospital admissions, standardised for the age distribution of the population, was 128 per 10,000 population [13]. In 2003, 9% of COPD patients was hospitalised [9]. The average length of hospital stay for COPD decreased from 15.2 days in 1998 to 10.4 days in 2007 [12]. The Confronting COPD survey indicated that in 2003, the average total direct costs per patient were €614 annually, of which 20% was spent on hospital admissions [9]. One-third of COPD patients report to have at least one comorbidity and 73% of patients use some (inhaled) medications for COPD [9].

### The organisation and funding of COPD care in England

In England, patients’ pathway in COPD care usually starts in primary care. General practitioners and practice nurses (although not always only dedicated to COPD) provide preventive care, diagnostics and treatment and follow-up care in stable and acute phases (exacerbations) of the disease. The number of FTE (Full Time Equivalent) general practitioners in 2005 was 55.7 per 100,000 population in the UK [14]. Eighty-six percent of patients stated that the general practitioner was the health professional they went to for the management of their COPD [8]. Only 14% received care from a respiratory physician [8].

COPD patients are offered several community services, for which most referrals are made by hospitals [15]. The community services are often led by specialist nurses [15]. In addition to general nursing services (medication support), practice nurses, community matrons or nurse specialists offer chronic disease management services (e.g. smoking cessation, education or long-term oxygen services) or acute management services (e.g. home visits or outpatient clinics) [15–17]. Services provided in the community are based secondary care (42%) or based in both secondary and primary care (41%) [15].

Patients can be referred to secondary care (hospitals) for specialist examination according to the NICE guideline [18]. This guideline also includes instructions on which patients should be treated in primary and secondary care (outpatient care) and when hospital admission should be considered. In addition to the inpatient treatment management of patients with acute exacerbations, hospitals also provide outpatient treatment and follow-up, diagnostic services and rehabilitation programmes. The available number of acute hospital beds in 2008 was 2.6 per 1000 population and 2.2 in 1995 [19]. The main professionals involved in hospital care for COPD are respiratory physicians and (specialised) nurses. The number of respiratory physicians per 100,000 population was 1.7 in 2007 [20]. Physiotherapists are also involved in COPD care, mainly in hospitals and community or hospital-based rehabilitation programmes.

A special service available in 44% of British hospitals is hospital-at-home for patients with a COPD exacerbation who require hospital admission [21]. This type of care was developed in the UK in the mid-1990s. It differs in various respects from other types of care. As said, hospital-at-home is intended for patients who would otherwise be or remain hospitalised. This means that the patient’s condition requires hospital admission. Care at home is similar to the care provided in hospital. Hospital-at-home is a nurse-led facility. Care is delivered by *specialised* respiratory nurses, mostly based (but not necessarily employed) in secondary care, under clinical responsibility of the respiratory physician in secondary care. Nurses visit the patient for several days (mean is 11 days) to monitor recovery and initiate the patient’s own disease management [22]. Approximately 30% of hospital admissions for exacerbations is considered eligible for hospital-at-home [21]. Costs of hospital-at-home are estimated to be lower than usual hospital care. Skwarska et al. [23] found the mean total health care costs of hospital-at-home to be £877 per patient, whereas usual hospital care costs £1753. In a Spanish hospital-at-home scheme, a difference in mean costs was found of €810 per patient [24]. These results should be interpreted with some caution, however, as outcomes of the cost-analyses are influenced by the decision as to which costs are incorporated and the design of the scheme. A schematic overview of the English care delivery pathway is offered in Figure 1A.

Care is funded by Primary Care Trusts (PCTs) which control 80% of the (tax-financed) budget for health care [25]. PCTs are responsible for the assessment of the health care needs of local communities and the commissioning of services from providers in primary care, secondary care, private providers and from community services [25]. In addition, PCTs employ staff to deliver care directly. This staff may be stationed in primary care practices, secondary care (hospitals), or other care facilities. This arrangement makes it possible for PCTs to base (respiratory) nurses in secondary care to provide hospital-at-home care for COPD exacerbations, but also to commission this service from hospitals or primary care facilities. In practice, most hospital-at-home schemes are hospital-based services with nurses working on an outreach basis.

### The organisation and funding of COPD care in the Netherlands

Care pathways of COPD patients in the Netherlands usually start in primary care. General practitioners are involved in the prevention, diagnosis and treatment of the disease in chronic and acute phases, and follow-up care. In 2009, the Netherlands had 42.5 FTE general practitioners per 100,000 population [26]. Some aspects of the care (e.g. education, inhalation instruction) are delegated to practice nurses. In 2007, approximately 62% of GP practices had practice nurses [27]; however, these nurses are often not dedicated to COPD only but also tend to patients with other chronic illnesses. Practice nurses mainly work in the general practice, but sometimes perform home visits as well (outreach).

Patients can be referred to secondary care facilities (hospitals) and professionals, according to guidelines [28, 29]. These guidelines describe which patients should be treated in primary or secondary care (outpatient care) and when hospital admission is indicated. The Confronting COPD survey revealed that, in 55% of patients, the general practitioner is the primary physician that treats the COPD patient, whereas for 42% this was the respiratory physician at the hospital [9]. Apart from inpatient treatment in acute phases of the disease, hospitals provide diagnostic facilities, specialist examination, prevention, treatment and follow-up (outpatient management) and outpatient rehabilitation programmes. Respiratory nurses are involved in some of these activities. The number of acute hospital beds was 3.5 beds per 1000 population in 1995 and 3 beds per 1000 population in 2008 [19]. The number of respiratory physicians in hospitals in 2011 was 2.7 per 100,000 population [personal communication]. Seventy-five percent of medical specialists are organised in partnerships and are paid fee-for-service [30]. The remaining 25% of respiratory physicians and all other hospital staff are salary-paid [30]. Physiotherapists involved in the treatment of COPD patients work in hospitals, in rehabilitation programmes (secondary care), and in community (maintenance) reactivation programmes. Community nursing is a facility available for patients in their home setting. Community nurses are employed by home care organisations [31] and are involved in general services such as nursing, medication supervision, washing and dressing. Community nurses with a focus on COPD/lung diseases also provide chronic management services like smoking cessation programmes and disease management [31]. Figure 1B shows an overview of the care delivery pathway for COPD patients in the Netherlands.

There are two separate insurance programmes for the funding of COPD care. Care provided by general practitioners, hospitals and physiotherapists is covered by the mandatory basic health insurance scheme and by voluntary, complementary insurance schemes [31]. Health insurers are responsible for the purchasing and funding of this care [31]. Community nursing is covered by the exceptional medical expenses scheme [31].

### Comparison of care delivery and explanation for hospital-at-home development in England

We find several common elements in the delivery of COPD care in England and the Netherlands. A first common element concerns the involvement of general practitioners, practice nurses or COPD nurse specialists in primary care. A second common element is the availability of inpatient and outpatient facilities in secondary care for diagnostics, prevention, treatment and follow-up care. However, England has a stronger focus on primary care, as illustrated by the higher number of FTE general practitioners per 100,000 populations in England (55.7 vs. 42.5) and the higher percentage of patients that is mainly treated by general practitioners. Furthermore, the percentage of patients annually admitted to hospital and the standardised hospitalisation figure is higher in England than in the Netherlands [8, 9]. Further research is required to explore these and other differences, as this is beyond the scope of this article. Nonetheless, it seems that the demand for hospital beds for COPD exacerbations is higher in England. A third common element is that both countries use guidelines to describe when patients should be treated in primary or secondary care, when referral is necessary and when admission to the hospital is indicated. A fourth common element is the presence of chronic management services in the community, mainly delivered by nurses. However, in England more diverse services are available at the community level. A final common element is that in both countries tasks and responsibilities have been delegated to nurses to relieve some of the pressure on general practitioners and hospital doctors.

A significant difference in the care delivery pathways between England and the Netherlands concerns the acute management of exacerbations that require hospital admission. In England a service for COPD exacerbations named hospital-at-home is available, whereas a comparable type of acute service to substitute hospital care when admission is indicated does not exist in the Netherlands. Why hasn’t any equivalent of hospital-at-home schemes for selected patients with a COPD exacerbation been developed in the Netherlands so far? We suggest three explanations: the differences in urgency for alternatives, differences in funding, and differences in the substitution of tasks to nurses. These three explanations do not exclude but complement each other.

#### Differences in funding

The first explanation concerns differences in the funding of COPD care. Whereas England has single-source funding, the Netherlands has a multiple-payer system with two separate insurance schemes. The single-source funding in England has much facilitated the development of integrated care, like hospital-at-home and staff working across institutional boundaries and across different levels of care. In the Netherlands, the institutional split between the basic health insurance scheme on the one hand and the exceptional medical expenses scheme on the other is hampering the coordinated delivery of COPD care and the development of intermediary services, such as hospital-at-home for acute COPD exacerbations. Hospital-at-home in England takes place under clinical responsibility of respiratory physicians in secondary care and works on an outreach basis from hospitals in the community, or it requires the involvement of community nurses. In the Netherlands, outreach activities in the community by hospitals, under responsibility of the hospital, are not reimbursed by the health insurance scheme. At the same time, however, the exceptional medical expenses scheme forbids community care providers to pay for care that takes place under responsibility of the hospital and that is covered by the hospital budget [31]. The example of hospital-at-home demonstrates how the co-existence of distinct financial flows in the Netherlands obstructs the integration of care. It is also important to note that, despite guidelines on who should be treated in primary and/or secondary care, there has always been some competition between hospitals, medical specialists and other providers (general practitioners, home care organisations) regarding the treatment of COPD patients. Physicians receive reimbursement for every patient they treat. Almost all patients are listed in a general practice but it is financially rewarding for respiratory physicians and hospitals to treat patients as well. Furthermore, the rivalry has been intensified by the fact that the payment of all providers has become more performance-related. Introduction of hospital-at-home in the Netherlands would have financial consequences for each provider. Fewer hospital admissions imply that hospitals would receive less funding as reimbursement is based on the number of registered admissions. This makes hospitals less willing to participate in outreach treatment for COPD-patients.

A related difference between England and the Netherlands is that the role of PCTs as funding organisation in England is stronger than the purchasing role of the health insurers in the Netherlands. PCTs have more leverage to initiate changes because they control almost the entire budget for health in their service area. In the Netherlands, health insurers have to compete with each other under the new Health Insurance Act, which may complicate the development of new forms of care (such as hospital-at-home). Furthermore, nearly all respiratory physicians in England are employed and paid by the NHS hospitals, whereas 75% of Dutch respiratory physicians are independent entrepreneurs. This results in a difference in power balance, interests and incentives for change. In hospitals where physicians are salaried employees, the hospital has more power than when physicians operate in entrepreneurial ventures.

#### Differences in urgency

The prevalence of COPD and the number of hospitalisations in England is higher than in the Netherlands, whereas England has fewer hospital beds than the Netherlands. Consequently, the pressure on the available beds is higher in England than in the Netherlands, especially during winters when the incidence of COPD exacerbations increases. These circumstances highlight how the urgency to develop alternatives for hospital admission and free hospital beds has been much higher in England than in the Netherlands.

#### Differences in the substitution of tasks to nurses

The third explanation concerns the role of nurses in health care provision. In hospital-at-home schemes, nurses supervise the treatment at home, which is a form of substitution. Both England and the Netherlands have delegated tasks from physicians to nurses, but in England the delegation started earlier than in the Netherlands, and also on a larger scale [32, 33]. In England the incentive to delegate was strong because of the low number of hospital doctors per 100,000 population and the government’s strategy to shift, as far as possible, health services from hospitals into the community (primary care and community care services) [34]. PCTs quickly recognised the cost-effectiveness of task substitution to nurses, and set up a large network of (specialised) nurses to perform tasks, often in the community [33]. The delegation of tasks (or task substitution) began later in the Netherlands [32] on the argument that expanding the medical role of nurses could impact the specific relationship between patients and nurses and the clearly defined caring function of nursing [33]. Furthermore, in the Netherlands physician scarcity—which is the main driver for substitution—was less urgent than in England.

## Lessons

In our comparison of the care delivery pathways of COPD patients in the Netherlands and England, we found not only common elements but also a significant difference: the presence of hospital-at-home for COPD exacerbations in England and its absence in the Netherlands. We suggested three complementary explanations for this remarkable difference. Table 1 shows that length of hospital stay is higher in the Netherlands than in England, while the Netherlands scores better with respect to the other variables. The third step in our analysis is to address the question whether the Netherlands could learn from England in order to reduce the length of stay, in particular with regard to hospital-at-home.

In England hospital-at-home has been shown to reduce the length of hospital stay: the average length of stay (which includes patients not eligible for hospital-at-home) in hospitals with hospital-at-home is 8 days vs. 9 days in hospitals without hospital-at-home [21]. In the Netherlands the average length of stay for COPD patients is still high with an average of 10.4 days. Although the reduction in length of stay reached by hospital-at-home in England is small, our suggestion is that the Netherlands can learn from this type of care. In England hospital-at-home for COPD exacerbations has been studied and described extensively. It has proved to be a safe alternative for traditional inpatient stay for selected patients, without negatively affecting patient outcomes [35]. In addition, patients accept this type of care and show great satisfaction [35].

In the Netherlands, the introduction of hospital-at-home for COPD exacerbations would perfectly fit the government’s strategy to delegate more tasks from hospital specialists and general practitioners to nurses. However, the introduction of hospital-at-home in the Netherlands has implications for the professionals involved. In order to realise a hospital-based outreach service, the available number of specialised nurses in hospitals must be increased. However, considering how the funding system prohibits hospitals from funding outreach care, the option of using more readily available (non-specialised) community nurses for the home visits should be considered, as has already been suggested by Davison et al. [36]. This development would expand nurses’ activities with the supervision of patients that would otherwise be hospitalised. Another alternative could be to use practice nurses employed by general practitioners. This would bypass some of the barriers of the funding system since the funding of hospital care and primary care are part of the same segment. Furthermore, clinical responsibility for hospital patients that are being treated at home needs to be dealt with in the current funding system. It should be further explored whether general practitioners are willing to take responsibility and whether this would be feasible, or whether it would be possible, within the current system, to retain clinical responsibility at the hospital.

The prospects of realising the introduction of hospital-at-home in the Netherlands have improved with the government’s strategy of shifting some forms of community nursing from the exceptional medical expenses scheme to the basic health insurance scheme. This may help bring about a better integrated system of health services for COPD patients. Another promising development concerns the introduction of a bundled payment system for chronic care [37]. The essence of this model is to pay an annual budget for the treatment of patients with a chronic disease such as COPD. The bundle-payment covers a wide range of services for COPD patient contracted and delivered by care groups (which are often general practitioners) in primary care, as well as one consultation of a respiratory physician in secondary care. The care group can deliver the services directly, or can contract other providers (e.g. physiotherapists) to do so. Unfortunately, the funding of inpatient care and outpatient care provided by the hospital is not (yet) included in this budget. Community nursing is not included in this budget either. Ideally, the chain funding would cover all types of care in primary care, secondary care and community care.

## Conclusion

This comparative study has demonstrated the differences and similarities in COPD care delivery pathways in the Netherlands and England. An important difference between both countries concerns the presence of hospital-at-home for COPD exacerbations in England. We have suggested three explanations for this difference. In England hospital-at-home for COPD exacerbations has proved to be a safe alternative for inpatient treatment, without adverse patient outcomes and with great patient satisfaction. We argue that the development of hospital-at-home COPD exacerbations should be seriously considered in the Netherlands to reduce length of hospital stay, which is significantly longer in the Netherlands than in England. Given some current changes in the funding of health care, the prospects for the introduction of hospital-at-home are improving.

## Figures and Tables

**Figure 1A.**
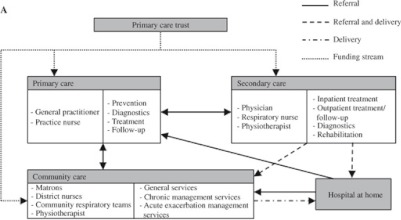
Care pathway in England.

**Figure 1B.**
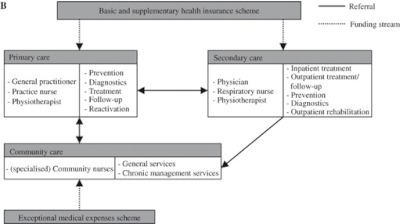
Care pathway in the Netherlands.

**Table 1.**
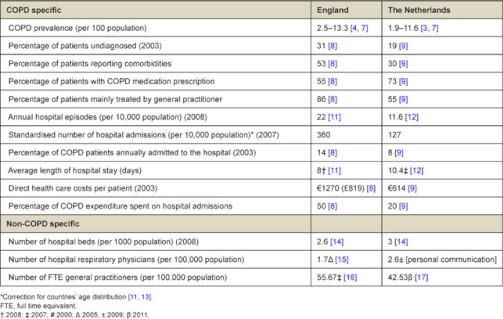
Statistics of COPD, related health care resources and health care costs with [reference]

## Reviewers

**David M. Mannino**, MD, Professor, Director of Graduate Studies, Masters of Science in Clinical Research Design; Director, Pulmonary Epidemiology Research Laboratory, Department of Preventive Medicine and Environmental Health, University of Kentucky College of Public Health, USA

**Felix S.F. Ram**, Clinical Pharmacologist (Respiratory), School of Health Sciences, Massey University, Auckland, New Zealand

**Geertjan Wesseling**, MD, PhD, Professor of Respiratory Medicine, Maastricht University Medical Centre, The Netherlands
